# Fatty acid oxidation in immune function

**DOI:** 10.3389/fimmu.2024.1420336

**Published:** 2024-06-27

**Authors:** Felicia Kemp, Erica L. Braverman, Craig A. Byersdorfer

**Affiliations:** Department of Pediatrics, Division of Blood and Marrow Transplant and Cellular Therapies, University of Pittsburgh School of Medicine, Pittsburgh, PA, United States

**Keywords:** fatty acid oxidation (FAO), immunometabolism, metabolic adaptation, metabolic dysregulation, immune cell differentiation, adoptive cellular therapies

## Abstract

Cellular metabolism is a crucial determinant of immune cell fate and function. Extensive studies have demonstrated that metabolic decisions influence immune cell activation, differentiation, and cellular capacity, in the process impacting an organism’s ability to stave off infection or recover from injury. Conversely, metabolic dysregulation can contribute to the severity of multiple disease conditions including autoimmunity, alloimmunity, and cancer. Emerging data also demonstrate that metabolic cues and profiles can influence the success or failure of adoptive cellular therapies. Importantly, immunometabolism is not one size fits all; and different immune cell types, and even subdivisions within distinct cell populations utilize different metabolic pathways to optimize function. Metabolic preference can also change depending on the microenvironment in which cells are activated. For this reason, understanding the metabolic requirements of different subsets of immune cells is critical to therapeutically modulating different disease states or maximizing cellular function for downstream applications. Fatty acid oxidation (FAO), in particular, plays multiple roles in immune cells, providing both pro- and anti-inflammatory effects. Herein, we review the major metabolic pathways available to immune cells, then focus more closely on the role of FAO in different immune cell subsets. Understanding how and why FAO is utilized by different immune cells will allow for the design of optimal therapeutic interventions targeting this pathway.

## Major metabolic pathways and available nutrient sources

Primary nutrient sources available to cells include sugars, amino acids, and fat; with both the availability of nutrients and the underlying cell phenotype strongly influencing engagement of specific metabolic pathways. Mechanistically, cells use both extracellular and intracellular machinery to sense local nutrient and energy levels, followed by upregulation of corresponding nutrient receptors and transporters (1). Glycolysis breaks down glucose, the predominant monosaccharide in the body, to produce energy in the form of adenosine triphosphate (ATP) and the reducing agent nicotinamide adenine dinucleotide (NADH), which in turn drives the electron transport chain (ETC). Pyruvate, a glycolytic pathway intermediate, can be further oxidized to acetyl-CoA, a primary fuel source for the tricarboxylic acid (TCA) cycle. Glycolysis can also generate ATP in the absence of oxygen, a process known as anaerobic glycolysis, with pyruvate bypassing the TCA cycle to be converted into lactate (2, 3). Amino acids (AAs), in contrast, predominantly contribute intermediates to the TCA cycle, which supplies reducing equivalents to drive ATP production. For example, glutamate undergoes oxidative deamination to produce α-ketoglutarate, which then feeds into the TCA cycle between isocitrate and succinate (4). However, not all AAs enter the TCA cycle at the same point, and convergence reactions are known to feed into pyruvate, acetylcholine, oxaloacetate, and α-ketoglutarate.

Lipids are high-energy compounds that produce more ATP per carbon molecule than glucose when fully catabolized (5). Fatty acids (FAs) are the principal components of dietary lipids, which are subsequently packaged into triacylglycerol moieties (TAGs) to be handled and transported throughout the body. TAGs can be synthesized in the intestine through the exogenous pathway or by the endogenous pathway in the liver. TAGs from the exogenous pathway are transported to the blood via the lymphatic system in the form of chylomicrons consisting of TAGs, cholesterol, and assorted proteins. Chylomicrons are taken up in the small intestine by lacteals, before migrating first to the mesenteric duct and then eventually the thoracic duct, from which they enter the circulation (6). The liver, in contrast, combines TAGs with lipoproteins to produce very low-density lipoproteins, which are then released into the circulation to be taken up by distal tissues and cells (5, 6).

## Immunometabolism

In some of the earliest studies on cellular metabolism, Warburg found that expanding tumor cells produced lactate from glucose, even in the presence of abundant oxygen, a process known as aerobic glycolysis (7). Strikingly, some of the earliest studies on T cell metabolism also pointed to *in vitro* use of aerobic glycolysis, particularly when T cells were stimulated through CD28 (8). Glycolysis being energetically less efficient, it was often wondered why T cells would favor this pathway during rapid cell proliferation, instead of using a more energy-rich form of nutrient like long-chain FAs. The main arguments supporting the adoption of glycolysis were that it could facilitate metabolic flexibility, encourage biomass generation, and produce additional intermediates necessary for cellular function (9–11). For example, glucose 6-phosphate, a glycolytic intermediate, could be readily shuttled through the pentose phosphate pathway (PPP) for nucleotide synthesis, while 3-phosphoglycerate could be siphoned off to produce serine and glycine, to promote glutathione production and purine synthesis. Further, glyceraldehyde 3-phosphate could be converted to glycerol 3-phosphate to create cellular lipids (12). Indeed, all of these pathways represent important steps in the generation of biomass needed to build new daughter cells. This initial interest in understanding how immune cells, and particularly T cells, make metabolic pathway decisions ultimately led to the now burgeoning field of immunometabolism (13, 14). Further, while the recent rebirth of interest in immunometabolism started with T cells and glycolysis, as we have begun to explore metabolism in different T cell subtypes (15), during *in vitro* versus *in vivo* activation (16), or in immune cell types beyond T cells, we have come to understand the significant complexity of immune cell metabolism. Indeed, these subsequent studies have identified roles for acetyl-CoA (17), acetate (18), glutamine (19), and fatty acid metabolism (20) in a variety of cells and at multiple stages of differentiation and activation. Many noteworthy reviews have been written highlighting general immunometabolism, contributions of select pathways, role of tissue-specific factors, and metabolism in defined subsets of cells (21–24). In this review, we will focus predominantly on the contributions of fatty acid metabolism, in particular fatty acid oxidation (FAO), and its impact on both innate and adaptive immunity.

## Fatty acid metabolism - an overview

Fatty acid metabolism can be grossly divided into anabolic and catabolic processes. Fatty acid synthesis (FAS), an example of an anabolic process, uses lipid byproducts to generate the building blocks of membranes, including those in the endoplasmic reticulum, Golgi apparatus, and intracellular organelles (25). Although this review will focus on lipid breakdown, because the products of FAS frequently supply the fuel for these oxidative pathways, therapeutic approaches that limit or drive oxidation must also consider the sources that drive this catabolic capability.

**Figure 1:**
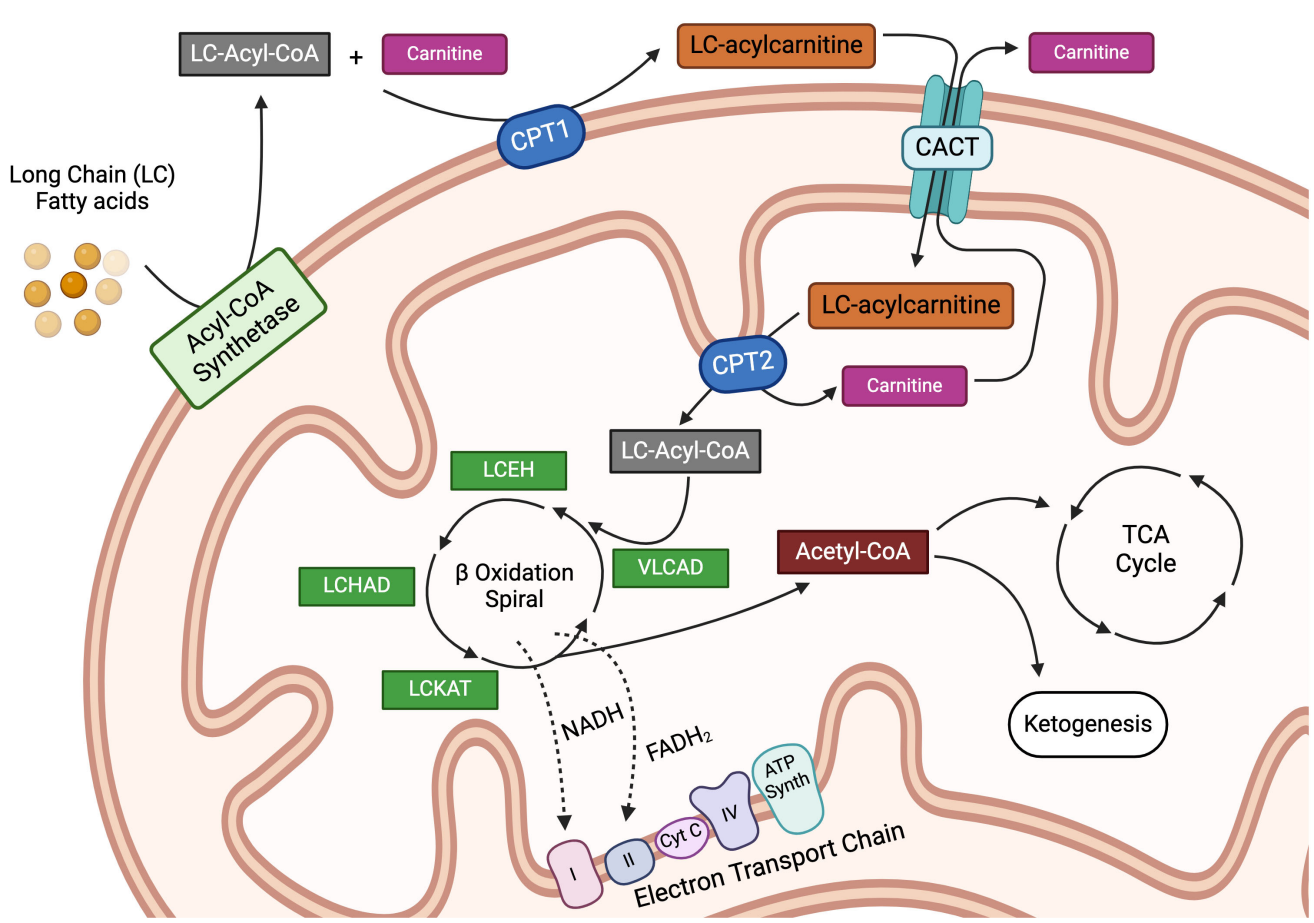
Intracellular transportation and oxidation of long-chain fatty acids. Long-chain fatty acids (LCFAs) are transported inside the cell via specialized transporters, where they are acted upon by a family of acyl-CoA synthetase enzymes to generate long-chain acyl-CoA (LC-Acyl-CoA) moieties. LC-Acyl-CoA is then conjugated to free carnitine by the enzyme carnitine-palmitoyltransferase 1a (CPT1a), in a conjugation reaction often delineated as the rate-limiting step in fatty acid oxidation. This conjugation reaction generates LC-acylcarnitine species, which can then be transported into the mitochondria by the carnitine-acylcarnitine translocase protein (CACT). Once inside the mitochondria, LC-acylcarnitine is deconjugated by the enzyme carnitine palmitoyltransferase 2 (CPT2) to free the LC-Acyl-CoA and carnitine, the latter of which is transported back out of the mitochondria in a loop known as the carnitine shuttle. Once inside the mitochondria, LC-Acyl-CoA enters into the beta-oxidation spiral, which is a 4-step series of dehydrogenation, hydration, oxidation, and finally thiolysis. Every cycle produces one molecule each of acetyl-CoA, NADH, and FADH2 while shortening the parent LC-Acyl-CoA by 2 carbons. This spiral continues until the LCFA is entirely broken down. The reducing agents, NADH and FADH2, produced during the β-oxidation reaction are used to fuel the electron transport chain, while the acetyl-CoA can either enter the tricarboxylic acid cycle as an intermediate or be used for ketogenesis. LCEH, long-chain enoyl-CoA hydratase; LCHAD, long-chain hydroxyl acyl-CoA dehydrogenase; LCKAT, long-chain 3-keto-acyl CoA thiolase, and VLCAD, very long-chain acyl-CoA dehydrogenase. Figure created using BioRender.

Most FAs in the Western diet, as well as those in the tissues of the body, are long-chain fatty acids (LCFAs), 12 to 22 carbon atoms in length, with the majority containing 16–18 carbons. Fatty acid catabolism breaks down these LCFAs for the provision of cellular energy (summarized in **Figure 1**). Long-chain fatty acid uptake is facilitated through a number of processes, while short and medium-chain FAs can diffuse directly into the cell. Albumin-bound FAs can be transported in pinosomes via micropinocytosis. Apolipoprotein B100 (apoB100), found on the external surface of Low-Density Lipoproteins (LDLs) and Very Low-Density Lipoproteins (VLDLs), binds to LDL and VLDL cell surface receptors, precipitating subsequent lipoprotein endocytosis (26). In addition, externally associated lipoprotein lipase (LPL) actively hydrolyzes triglycerides from chylomicrons, LDLs, and VLDLs, which then enter the cell through one of six fatty acid transporter proteins (FATP) and/or CD36 (27–29). Extracellular free fatty acids can also traffic either directly into the cell or via FATPs (26, 30). Once inside the cell, LCFAs are activated by an isoform of acetyl-CoA synthetase and made available for storage or subsequent oxidation (31). In FAO, acyl-CoA moieties are conjugated to carnitine by the enzyme carnitine palmitoyltransferase 1A (CPT1A), with subsequent transport of acylcarnitine species across the mitochondrial membrane by carnitine acylcarnitine translocase (CACT) in exchange for a free carnitine molecule, a process referred to as the “carnitine shuttle.” Next, the intramitochondrial enzyme, carnitine palmitoyltransferase 2 (CPT2), decouples the acylcarnitine back to a long-chain acyl-CoA (LC-acyl-CoA) species (32), freeing carnitine to be transported back to the cytosol. Once inside the mitochondrial matrix, these LC-acyl-CoAs are repeatedly broken down at the β carbon position, in an enzymatic spiral, producing single two-carbon acetyl-CoA species during each turn of the cycle and continuing until the FA has been completely consumed (3–5). The resulting acetyl-CoA moieties can then enter the TCA cycle or become part of the ketone synthesis pathway. This comprehensive breakdown of fat through the process of FAO provides a rich source of cellular energy. Moreover, several studies in recent years have found that fatty acid metabolism plays a major regulatory and/or differentiation role in immune cells, having been implicated in modulating both pro and anti-inflammatory immune responses. This exciting area of ongoing research, termed “bidirectional metabolic signaling,” examines the crosstalk between metabolism and signal transduction in the orchestration of immune responses (33). Here, we will explore the role of FAO in the major immune cell subsets and, when possible, will highlight this concept of bidirectional signaling.

## FAO in specific types of immune cells

### Innate lymphoid cells

Innate lymphoid cells (ILCs), the innate analog to T cells in both humans and mice, includes NK cells, ILC1s, ILC2s, ILC3s, and Lymphoid Tissue Inducer (LTi) cells (34). ILC1s functionally complement CD4+ T helper type 1 (Th1) cells, ILC2s complement Th2 cells, and ILC3s complement Th17 cells. NK cells act much like their cytolytic CD8+ T cell counterparts, and LTi cells are responsible for structural support and the development of secondary lymphoid organs (SLOs) (34, 35). As a greater appreciation for the contributions of ILCs to barrier immunity and tissue homeostasis emerges, so does a broader understanding of their metabolic requirements. Most ILCs are tissue-resident, with their metabolic profiles reflecting nutrient availability in their local environments (36). In concert with this idea, recent transcriptional murine studies have revealed distinct lipid metabolism profiles for each ILC subset. As an example, the ability of ILC2s, the largest population of tissue-resident ILCS in the lungs, to instigate inflammation has been linked to their metabolic profile (37), with a transcriptome indicating high engagement of FA metabolism (38). More recent work in a model of allergen-induced airway inflammation found that ILC2s take up external lipids, such as the long-chain FA palmitate, and store them in the form of lipid droplets. These fatty components are later oxidized in a process initiated by IL-33 and mediated by peroxisome proliferator-activated receptor-γ (PPAR-γ) and diacylglycerol O-Acyltransferase 1 (DGAT1), leading to expanded numbers of pro-inflammatory ILC2s in the lungs. Switching mice to a ketogenic diet ablated this lipid accumulation and resolved the airway inflammation. In these studies, a ketogenic diet was defined as a diet composed of a 4:1 fats to carbohydrates ratio, whose purpose was to reduce glucose availability to subject animals (39). In another example of FAO dependency, under conditions of vitamin A deficiency, ILC2s maintain IL-13 production during helminth infections by increasing their uptake of environmental FAs and utilizing FAO, thus preventing tissue barrier compromise in mice (40). Finally, a lack of autophagy in activated ILC2s during allergic asthma impaired FAO utilization and strikingly promoted glycolysis instead. This shift in nutrient dependency impaired ILC2 homeostasis and Th2 cytokine production, inhibiting the development of ILC2-mediated lung inflammation (41). Notably, this metabolic reprogramming (FAO to glycolysis) was also associated with an accumulation of dysfunctional mitochondria and excessive production of reactive oxygen species (ROS).

Recently, it was shown that intracellular Ca2+ elevation in ILC2s, via Ca2+ release-activated Ca2+ (CRAC) channels, plays a prominent role in their capacity for metabolic adaptation. Blockade or genetic ablation of the CRAC channel, or its components Orai1 and Orai2, inhibited FAO and disrupted mitochondrial homeostasis, subsequently upregulating ROS production. These changes, in turn, diminished ILC2 effector function and cytokine production, ameliorating the development of ILC2-mediated airway inflammation in animal models. Similar findings were demonstrated in human ILC2s, where blockade of Orai1 and Orai2 prevented airway hyperreactivity in humanized mice (42).

Early work made a case for murine NK cell reliance on glycolysis, with partial regulation by amino acid intermediates (43, 44). However, more recent reports demonstrate that NK cells activated upon acute retroviral infection increase the expression of CD36, a transporter suggested to mediate FA uptake. Activated NK cells, also demonstrated enhanced flexibility to utilize FAs (including linoleic, palmitic, and oleic acid) as energy sources, with increased FA energy generation correlating with augmented cytotoxicity (43). Conversely, NK cells unable to generate energy from FAs exhibited a decreased migratory capacity, implying that inventions that boost FA processing and oxidative capacity might be prime therapeutic targets to maximize NK cell function (45).

### Dendritic cells

Dendritic cells (DCs) consist of myeloid and lymphoid subtypes, including the conventional DC subsets (cDC1, cDC2), recently identified human cDC3 cells, as well as plasmacytoid DCs (pDCs), inflammatory DCs, and Langerhans cells (46–48). DCs operate at the junction of innate and adaptive immunity by processing and presenting peptide antigens to cognate T cells while providing fate-determining co-stimulation signals and activation associated cytokines. Conventional DCs specialize in activating naïve T cells, with cDC1s classically presenting exogenous peptides on MHC-I molecules to CD8+ T cells, where they generate cytolytic T cell responses capable of targeting both tumor and virally-infected cells. Conversely, cDC2s activate CD4 T cells via MHC-II antigen presentation to drive Th2 and Th17-mediated responses to parasites, bacteria, fungi, and allergens (49, 46, 47). cDC3s share phenotypic characteristics with cDC2s. showing comparable T cell stimulation *in vitro* and greater Th17 polarization by cDC3s. However, cDC3s arise from a unique Ly6C+ monocyte-DC progenitor, as opposed to the common DC progenitor giving rise to cDC1/2s, and thus represent a divergent lineage. cDC3 cells have not been metabolically profiled to date (50). pDCs do not typically participate in antigen presentation; however, these “steady-state” cells produce high quantities of anti-viral type I interferons, which have been shown to contribute to autoimmunity (47, 49, 51). Traditionally, DC subsets were distinguished by transcriptional variability, development status, extracellular phenotype, and functional differences. However, recent advances in metabolic profiling have provided additional insights into variations within these specific DC subtypes. For example, although murine bone marrow-derived DCs (BMDCs) can give rise to both cDC1 and cDC2 subsets, pharmacologic inhibition of both FAO and AMP-activated protein kinase (AMPK) skewed differentiation towards the cDC2 phenotype. In contrast, reducing ROS in developing DCs increased the proportion of cDC1 cells (52, 53).

The effects of fatty acid exposure have been studied in parallel in both murine and human DCs. In both instances, DCs were derived from progenitor populations and differentiated *in vitro*. Murine BMDCs were differentiated *in vitro* with granulocyte-macrophage colony-stimulating factor (GM-CSF) and/or Fms-related tyrosine kinase 3 ligand (FLT3L), alongside extracellular FA exposure during development and activation. Monocyte-derived DCs (moDCs) were generated from human peripheral blood mononuclear cells using GM-CSF and interleukin 4 (IL-4) (54–56). Saturated and polyunsaturated fats differed in their impact on DCs, with saturated fats activating toll-like receptors (TLRs) to induce expression of costimulatory and MHC molecules, as well as pro-inflammatory cytokines. In contrast, polyunsaturated fatty acids (PUFAs), such as the long chain FA docosahexaenoic acid, inhibited lipopolysaccharide (LPS) driven pro-inflammatory signaling and prevented DC activation (57). Activation of TLRs also triggered *de novo* FAS and lipid droplet formation while down-regulating FAO. Together, these actions equip DCs with the appropriate energy reserves for activation and subsequent T-cell priming (58). Conversely, in some contexts, excessive lipid accumulation perpetuates DC *dysfunction*, a process implicated in some forms of impaired T-cell activation (59). In one specific example, tumor-associated DCs in lung cancer patients accumulate oxidized forms of truncated lipids, changes which render DCs less effective at activating cytolytic T cells, thus linking a metabolic effect emanating from the cancer to a decrease in T cell responses (60).

In addition to activating T cells, DCs also promote tolerance by influencing regulatory T cells (Treg). Tolerogenic DCs (tolDCs) maintain central and peripheral T cell tolerance through several mechanisms, including induction of anergy, deletion of antigen-specific T cells, and promotion of Treg differentiation. Tolerogenic DCs are characterized by increased anti-inflammatory markers (IL-10 and TGF- β) and decreased pro-inflammatory cytokines, with a developmental state described as semi-mature (61). Some cancers demonstrate higher rates of tolDCs, leading to increased Treg levels and a pro-tumor phenotype. It has also been shown that the short-chain FA butyrate, a bacterial metabolite, inhibits histone acetylation on murine BMDCs and induces Treg differentiation (62). In human DCs, butyrate induced polarized naïve CD4+ cells into IL-10-producing type 1 regulatory T cells (Tr1) through DC induction of the enzyme retinaldehyde dehydrogenase 1. This induction relied on G protein receptor 109A signaling together with inhibition of histone deacetylation (63). Another study demonstrated that melanoma tumors could activate Wnt5a-Beta-catenin and PPAR-γ in DCs utilizing a paracrine response pathway. These signaling cues then upregulated FAO, in the process generating a pool of Treg and inducing a tolerogenic state. Conversely, interrupting the PPAR-γ/FAO pathway increased the efficacy of PD-1 blockade and enhanced anti-tumor responsiveness (64).

Metabolic modulation via metformin treatment has also shown promise in inflammatory diseases by inducing tolDCs and driving subsequent Treg generation. Interestingly, the proposed mechanism in these studies is the promotion of glycolysis and inhibition of FAO, resulting in lipid accumulation in DCs treated with metformin (Met-DCs) and stimulated by LPS (65). Through untargeted metabolomic analysis, Met-DCs were shown to sustain higher levels of FAs with reduced FAS enzymes (e.g. acetyl-CoA carboxylase 1 (Acc1)) and lower metabolic intermediates, including citrate. Met-DCs also had reduced levels of carnitine and CPT1a, both of which facilitate transportation of LCFAs into the mitochondria. Thus, Met-DCs exhibited less FAO and a lower Oxygen Consumption Rate (OCR), consistent with decreased levels of oxidative phosphorylation (OXPHOS) and subsequent accumulation of lipids and free FAs. Functionally, metformin reduced the production of several proinflammatory cytokines while simultaneously increasing IL-10 secretion and expression of the immunomodulatory molecules ICOSL and PD-L1/2, together facilitating increased Treg production (65).

It was recently shown that murine tolDCs, generated through ablation of the transcriptional regulator Nuclear Receptor Corepressor 1 (NCoR1), meet a portion of their increased energy needs through enhanced FAO and a subsequent increase in oxygen consumption. Inhibition of β-oxidation with etomoxir, in combination with the administration of a HIF-1alpha inhibitor, compromised the tolerogenic transcriptional signature of these cells, diminishing production of the regulatory cytokines IL-10 and IL-27 (66). These decreases in regulatory cytokines influenced polarization of co-cultured naïve CD4+ T helper (Th) cells, pushing them towards a Th1 phenotype and away from Treg differentiation. Importantly, these findings were validated in primary human moDCs and together demonstrate that NCoR1-mediated control of glycolysis and FAO fine-tunes the balance between immune tolerance and inflammation in both murine and human DCs (66).

Dendritic cells are amongst a subset of cells known as mononuclear phagocytes (MPs), which play a vital role in the host immune defenses against cancer. MPs include DCs, tissue-resident macrophages, and circulating monocytes. Recent studies indicate that increased lipid accumulation in tumor-associated MPs tilts these immune cells away from utilizing glycolysis and towards mitochondrial FAO as a primary energy source. This phenomenon has been shown both in tumor-bearing mice and in patients with head-and-neck, non-small-cell lung, and renal cancers. as a consequence of increased FAO, tumor-infiltrating DCs differentiate toward a tolerogenic phenotype (tolDCs), with low antigen-presenting capacity and poor T cell priming (67–69). At the same time, tumor-associated macrophages (TAMs) are polarized into M2-like TAMs that suppress inflammation and down modulate T cell function (70, 71). Intriguingly, delivery of nanoparticles impregnated with both a viral RNA analog and the FAO regulator, cryptotanshinone (CTS), pushed tumor-infiltrating MPs towards an M1 macrophage repolarization and DCs from a tolerogenic to immunogenic differentiation. These innate cell changes subsequently increased pro-inflammatory cytokine secretion and recruitment of CD8+ T cells to the tumor microenvironment (TME) while enhancing susceptibility to anti-PD-1 mediated immune checkpoint therapy in multiple checkpoint blockade-resistant mouse models (72). Thus, inhibition of lipid metabolism, directed specifically towards MPs, could represent a promising strategy to reboot immune cell activation and potentiate the existing power of conventional immunotherapies.

We pause here to acknowledge the unique and important context of the TME, where multiple and often simultaneous challenges and changes impact the subsequent ability of immune cells to elicit potent anti-tumor responses. These factors include a limitation of nutrients, a lack of oxygen, competition for scarce resources, acidity, and saturation with immunosuppressive factors (73, 74). For this review, we will focus our discussion of the TME solely on points relating to lipid metabolism and FAO. For a broader view of immunometabolism within the TME, readers are directed toward many excellent recent reviews on this topic (75–77).

### Macrophages

Macrophages are a heterogeneous population of multi-functional innate immune cells capable of phagocytosis. Macrophages maintain organismal homeostasis by clearing cellular debris and scavenging iron while concomitantly influencing adaptive immune responses. They are broadly classified into tissue/resident and circulating (bone marrow-derived) subsets. Tissue macrophages are established during embryonic development while circulating macrophages originate from monocytes coursing through the blood (78).

Macrophages also exist in a resting or activated state, with activated macrophages further divided into M1 (so-called “classical” or “inflammatory” macrophages) and M2 (aka “alternative” or “reparative”) subtypes. Important for this review is the concept that M1 and M2 macrophages display distinct metabolic profiles that, in some cases, directly correspond to their disparate phenotypes and functionality (79, 80). Newer terminology seeks to expand these distinctions and groups macrophages based on their roles in host defense, wound healing/repair, and immune regulation (78, 81), which we will incorporate into our discussion. Broadly speaking, inflammatory M1 macrophages rely predominantly on glycolysis and glucose-driven pathways while reparative M2 macrophages predominantly utilize FAO and drive OXPHOS.

Classically activated (M1) macrophages arise following exposure to interferon-γ (IFNγ) and/or tumor necrosis factor (TNF), express high levels of cytokines IL-1, IL-6, and IL-23, and are adept at producing ROS, all traits as possible which make them highly effective killers during infection or in response to cancer. M1 macrophages, in both mice and humans, rely upon glucose-based metabolism, particularly the PPP, to produce nucleotide precursors and NADPH (82, 79). In these cells, ROS are generated via mitochondrial respiration and the activity of inducible nitric oxide synthase (iNOS), which converts arginine to nitric oxide (NO) (79).

Reparative M2 macrophages, in contrast, do not present antigen, produce pro-inflammatory cytokines, or generate oxygen radicals. Instead, M2 macrophages participate in wound healing and tissue repair following trauma; predominantly by rebuilding the extracellular matrix and working to offset effects from damaging pro-inflammatory cytokines through the production of counteractive polyamine compounds (81). M2 macrophages also utilize hydrolase arginase-1 to break down arginine, limiting its concentration in the local milieu and thereby depriving T cells of this vital nutrient and subsequently reducing T-cell proliferation (83). Both human and murine M2 macrophages have increased FAO, OXPHOS, and lipolysis compared to M1s. FAO is mediated by the PPAR family members PPAR-γ and PPAR-δ in M2s to drive OXPHOS and meet their energetic demands (14, 79, 83). Notably, macrophages are highly plastic cells, capable of rapidly polarizing in response to extrinsic signals, and significant work has been done to understand the mechanisms by which metabolic programming determines macrophage polarization in the hopes of strengthening therapeutic interventions (71, 84).

Infecting mice with the pathogen *Mycobacterium tuberculosis* (Mtb) promotes FAO in macrophages during lung infection (85), in some cases relying on the expression of the anti-inflammatory regulator Dual Specificity Phosphatase 5 (DUSP5) downstream of the TLR2-MAPK signaling pathway (86). Silencing RNAs (siRNA) specific to DUSP5 increased free FA content and triglyceride levels in macrophages but repressed expression of FAO-associated transcripts and enzymes, including CPT1A and PPAR-α (87). DUSP5 knockdown also reduced expression of proinflammatory cytokines IL-1β and IL-6 and inactivated NF-κB signaling in BCG-infected macrophages. Importantly, direct pharmacologic inhibition of FAO showed similar effects (suppressed IL-1β and IL-6 expression), with a decrease in pathologic lung damage in animal models of infection (87). Together, these data indicate that FAO reprogramming in lung-associated macrophages occurs via a DUSP5-mediated mechanism in response to BCG infection and offers both DUSP5 modulation, and FAO inhibition, as potential therapeutic avenues to modify the severity of sequelae associated with Mtb infection.

Functionally targeting macrophage metabolism has also shown particular promise in anti-tumor therapies. Macrophages infiltrating into tumors (i.e. TAMS) have diverse roles, with M1 TAMs promoting anti-tumor activity by inducing antibody-dependent cell-mediated cytotoxicity and facilitating CD8+ directed cytolytic killing by presenting tumor-specific antigens (88, 89). In contrast, M2-polarized TAMs create a pro-tumorigenic state by enforcing immune-mediated tolerance. The immunosuppressive functionality of TAMs is modulated by unsaturated LCFAs, as demonstrated in a recent murine study where oleate-treatment tolerized TAMs (90) by enhancing PPAR-γ signaling and upregulating FAO. Liu et al. investigated the oncoprotein S100A4, found in stromal cells and the TME, and its role upstream of PPAR-γ in TAM polarization in murine models. Macrophages lacking S100A4 were deficient in CD36, the aforementioned fatty acid transporter, and found equally unable to upregulate FAO during macrophage polarization, thus retaining a pro-inflammatory anti-tumor phenotype (91). Therefore, preventing the induction of S100A4 offers promise in treating cancers by reprogramming macrophage metabolism towards one favored by M1 macrophages and thus rebalancing immune reactions within the TME.

In macrophages, CD40 signaling also drives pro-inflammatory and anti-tumorigenic polarization through a mechanism suggested to rely on metabolic programming but which has yet to be fully articulated (92). Recent work in mice has shown that CD40 activation triggers FAO, which supports histone acetylation and subsequent epigenetic reprogramming of pro-inflammatory genes and anti-tumorigenic phenotypes. Inhibition of OXPHOS with rotenone or oligomycin impaired CD40-mediated pro-inflammatory gene expression, while anti-CD40 agonist treatment induced the opposite effect, increasing FAO-dependent OCR and inducing proinflammatory gene expression in macrophages pre-loaded with palmitate. Importantly, these changes could be abolished by treatment with the CPT1a inhibitor, etomoxir. In line with these mechanistic data, genetic ablation of essential metabolic enzymes, including CPT1a and ATP citrate lyase, abolished antitumor responses mediated by anti-CD40 treatment (93). Together, these data indicate that metabolic modulation in macrophages, including reprogramming of FAO, could serve a vital therapeutic preconditioning role in TAMs prior to agonistic anti-CD40 treatments.

### B cells

B cells, originating from the bone marrow, are responsible for driving the humoral arm of adaptive immunity. Naïve B cells differentiate into diverse subpopulations that control infection and tumor growth chiefly through the production of antibodies (94). Naïve peripheral B cell populations are typically divided into B1, B2, and B regulatory cells (Breg) subsets. B1 cells develop in the fetal liver and are considered a component of the innate immune system. While they persist into adulthood, their frequencies are low in both mice and humans. Their primary function, observed in mice, is to produce highly reactive, low-affinity natural immunoglobulin (95). Murine B1 cells predominantly reside and self-renew in the lipid-rich peritoneum and, perhaps because of this location, are metabolically distinct from B2 cells. B1 cells utilize glycolysis and fatty acid synthesis, importing exogenous lipids from the local environment to generate intracellular lipid droplets. Whether B1 cells subsequently use their fat reserves for energy production and FAO is unclear. What is known is that murine B1 cells are metabolically inflexible in the absence of autophagy, as B1 cell numbers are reduced, fat metabolism transcripts are down-regulated, and compensatory uptake in glucose is minimized following conditional deletion of the autophagy gene Atg7 (96). As human B1 cells have been hypothesized to be the cellular source of chronic lymphocytic leukemia, the most common form of leukemia in adults, understanding their metabolism in future studies could inform therapeutic approaches in targeting these cells (97).

B2 cells, also known as conventional B cells, include plasmablasts, plasma cells, naive B cells, and memory cells, the latter of which arise from the naïve population following antigen encounter. All B cell subtypes are capable of driving antibody production, while conventional B cells also serve as professional antigen-presenting cells (APCs) akin to DCs. Surface immunoglobulin on naïve B cells helps to select specific antigens, in mice and humans alike, which are then internalized and truncated into specific peptides, followed by subsequent presentation to T cells. It has been suggested that naïve murine B cells have low energy requirements supported by FAO (98–100), while activated B cells in mice increase glucose transport and oxygen consumption, including the shuttling of glucose intermediates into the PPP. Blocking glycolysis with 2-deoxy-d-glucose (2DG) curtailed B cell differentiation while maintaining their potential for self-renewal. Similarly, limiting glucose availability during *in vitro* antibody generation severely reduced class switching, with limited production of IgG1 antibodies in murine splenic and human peripheral B cells (101–103).

Formation of a germinal center (GC), where B cells interact with specialized T follicular helper (TFH) cells, is an integral part of generating high-affinity antibodies, as the GC is the localized site where somatic hypermutation and affinity maturation of the antibody response occurs. Importantly, dysregulation of these processes, particularly those resulting in the generation of antibodies with high-affinity to self-antigens, is thought to drive autoimmune diseases including systemic lupus erythematosus (SLE) and rheumatoid arthritis (104). Initial metabolic analyses, using murine GC B cells derived or manipulated *in vitro*, suggested that high levels of cellular glycolysis were essential for GC B cell generation and maintenance (105). Recently, however, when *in vivo* murine GC B cells were interrogated directly *ex vivo*, they were instead found to rely heavily upon FAO. ^13^C16-palmitate labeling of GC B cells showed that the majority of the acetyl-CoA pool originated from FAO (106). Moreover, RNA-sequencing of resident GC B cells showed that glycolysis-related HIF-1a target genes, originally increased in GC B cells propagated *in vitro*, were actually low in cells taken directly *ex vivo*. This finding led the authors to conclude that GC B cell gene expression did not demonstrate an environment of “functional hypoxia,” challenging the dogma that hypoxic conditions directed a glycolytic profile in GC B cells (106). Indeed, multiple studies have supported the idea of GC B cells being reliant on FAO, including those indicating that murine GC B cells rely heavily on autophagy to supply FAs for subsequent oxidation (107, 108).

Consistent with a role for fat metabolism in GC B cells, previous studies identified an association between the sterol regulatory binding protein (SREBP) pathway and humoral immune responses following vaccination in humans. More recently, B cell-specific deletion of SCAP, an essential regulator of SREBP signaling, decreased antibody responses and impaired GC formation in mice. Genes that encode long-chain acyl-CoA synthetases (ACSLs) - enzymes responsible for generating fatty acyl-CoA moieties from free fatty acids, play a necessary role in lipogenesis, β-oxidation, and protein acylation. Mechanistically, stimulation with either LPS or agonistic CD40 antibody significantly increased expression of Acsl3 and Acsl4 in WT but not SCAP-deficient B cells (109). However, rather than directly affecting FAO, SCAP deficiency suppressed regulation of genes involved in sterol biosynthesis, including critical enzymes such as 3-hydroxy-3-methylglutaryl coenzyme A reductase. Targeted metabolomics on activated SCAP-deficient B cells demonstrated many altered metabolites, most of which represented members of the ceramide and sphingolipid families (109). Thus, SREBP signaling is essential for metabolic reprogramming in GC and activated B cells. However, the degree to which these pathways directly impact FAO requires further study.

As noted above, autoreactive B cells are pivotal in the development of SLE. In animal models of this disease, CD36-mediated lipid uptake by B cells enhances mitochondrial OXPHOS. In a recent report, inhibition of FAO reduced autoreactive B cell responses and ameliorated disease in SLE mice. Mechanistically, FAO in the autoreactive B cells was shown to be dependent upon acetylcholine (ACh) donated from local splenic fibroblastic reticular cells (FRCs). Ach donated by adjacent FRCs promoted lipid influx into B cells through CD36, facilitating FAO and subsequent generation of autoreactive cells (110). These data suggest that a novel function of splenic FRCs is to control lipid metabolism and thus orchestrate B cell differentiation and maturation. They further identify splenic FRC-derived ACh as a key factor in controlling autoreactive B cell responses in SLE by indirectly controlling local nutrient availability.

### T cells

Thymus-dependent cells, aka T cells, originate from precursors that migrate from the BM to the thymus, in both mice and humans, for the final steps of maturation. Two T cell lineages, encoding different antigen receptors, emerge from a common CD4/CD8 Double Negative (DN) progenitor thymocyte population. These lineages are designated as γδ T cells (the minority population) and αβ T cells (the majority population of circulating cells) based on T Cell Receptor (TCR) chain expression. DN γδ T cells migrate directly from the thymus to the periphery, predominantly in the skin, intestine, and the lungs, whereas DN αβ T cells remain in the thymus and undergo further development (94, 111). While metabolic research has found correlations between lineage, functional commitment, and anti-tumor activity in γδ T cells, these studies have not revealed an explicit use of FAO; although further investigation is warranted (112, 113).

Double negative αβ T cells which remain in the thymus undergo a series of complex differentiation and selection steps before eventually emerging into the circulation and moving to secondary lymphoid organs (SLOs) as mature CD4+ and CD8+ T cells (94). During this thymic differentiation, a careful interplay of metabolic shifts occurs. In mice, early engagement of the Notch and phosphatidylinositol 3-kinase (PI3K) signaling pathways, coupled with interleukin-7 (IL-7) signaling, promotes glycolysis and glutaminolysis in double-negative thymocytes (DN) (114). However, as CD4/CD8 DN differentiation continues into stages that engage signaling through the pre-T cell receptor (TCR) complex, thymocytes begin to upregulate mitochondrial metabolism with an increasing reliance upon OXPHOS. This persists in naïve T cells, which traffic to lymph nodes and from which they then egress by following a chemotactic gradient of sphingosine-1-phosphate (S1P). This lipid-derived compound binds to a family of S1P receptors (e.g. S1P1R) to promote survival and in some cases FAO in animal models (115).

A role for FAO during thymocyte development is further implicated by the finding that peroxisome proliferator-activated receptor–δ (PPAR-δ), a transcription factor responsible for promoting expression of genes involved in FAO, is required for proper αβ T cell development. Deletion of PPAR-δ, specifically at the DN3 and DN4 stages of murine thymocyte maturation, reduces thymus cellularity as a result of impaired proliferation, causing subsequent peripheral lymphopenia in PPAR-δ KO mice (116). In support of FAO importance, when apoptosis-inducing factor (AIF), a Janus protein implicated in maintaining mitochondrial ETC function, is ablated in early murine T cell progenitors, the ensuing disruption in OXPHOS is compensated for by upregulating key FAO-associated enzymes including CPT1, acyl-CoA dehydrogenase long chain (ACADL), and pyruvate dehydrogenase kinase 4. Accordingly, AIF KO thymocytes had increased respiratory capacity and an enhanced sensitivity to etomoxir in the presence of the FA substrate palmitate (117). Thus, FAO is a critical metabolic process in T cells at early stages of development and differentiation, with disruptions having potential long-term consequences for peripheral T cell health and function.

Mature αβ T cells are broadly classified into two further categories based on the cell surface expression of CD8 and CD4 and their associated ability to recognize peptides presented by MHC Class I and II molecules, respectively. Naïve αβ T cells encounter their cognate antigen alongside cytokine and co-stimulatory signals to become activated, proliferate, and undergo differentiation. In the classic paradigm, CD8+ T cells differentiate into cytolytic effector T cells (Teff) capable of targeting and killing virally infected cells and tumors. As cytolytic responses resolve, a small subset of these cells transition into memory cells, poised to reactivate and expand upon re-encountering their antigen (118). These resting memory cells take on a more stem-like phenotype, allowing them to persist in the body for extended periods of time (119, 120).

Classically, naïve CD4 T cells differentiate upon activation into T helper cell subsets including Th1, Th2, Th17, T follicular helper, and regulatory T cells, often based on cytokine signals delivered by the surrounding milieu (94). Coincident with changes in their functional phenotype are metabolic reprogramming events, studied in both human and murine cells. As noted above, naïve T cells are largely quiescent while residing in SLOs, where they predominantly rely on catabolism and oxidative phosphorylation for cellular maintenance, including a notable dependence on FAO (121). In contrast, early studies suggested that activated T cells adopt a glycolysis-centric phenotype, with a subsequent return to oxidative metabolism and FAO in memory T cells (14, 22). This paradigm has been challenged in mouse models in recent years with the demonstration that T cells activated *in vivo* do not cycle carbons from glucose into lactate but rather transition these glucose carbons into intermediates of the TCA cycle (16). Further, activated T cells recovered on day seven following allogeneic transplantation, during an early phase of activation, demonstrate a marked increase in FAO (122). Thus, there are clear examples where activated Teff, particularly those *in vivo*, upregulate metabolic pathways beyond glycolysis. Some of the discrepancies with earlier mouse studies can be accounted for by different adaptations made by T cells based on their surrounding environment, which differs enormously based on whether the T cells are activated *in vitro* versus *in vivo* (16).

Interestingly, the cellular energy sensor AMP-activated protein kinase (AMPK), a known driver of oxidative metabolism and mediator of FAO, has also been implicated in promoting Th1 versus Th2 differentiation. Increasing AMPK activity in primary human CD4+ T cells pushed these cells towards an exaggerated Th1 phenotype (with increased interferon gamma (IFNγ) and TNF)) while also downregulating the production of the Th2-associated cytokines IL-4 and IL-5. Furthermore, this polarization occurred in both bulk and previously differentiated populations (123). However, work with murine T cells lacking AMPK demonstrated no difference in FAO when activated *in vivo*, despite an appreciable difference in oxidative metabolism (124). These data suggest that despite a known role for AMPK in driving FAO in other cells and tissues, its role in Th differentiation appears independent of FAO metabolsm.

Another mechanism for reprogramming T cells towards FAO relies upon the ligation of PD-1. Indeed, PD-1 binding in activated human T cells downregulates both glycolysis and glutaminolysis while significantly increasing their SRC (125). Mechanistically, PD-1 signaling upregulated levels of CPT1A and adipose triglyceride lipase while also promoting endogenous fatty acid release from intracellular stores to fuel β-oxidation (125). A parallel study found that T cells recovered following allogeneic bone marrow transplantation increased both PD-1 expression and ROS levels. Blocking the PD-1 signal in the GVHD environment, using anti-PD-1 antibodies, reduced both total and mitochondrial ROS production in murine and human T cells and simultaneously increased GVHD severity in animal models. Intriguingly, etomoxir treatment ameliorated PD-1-driven increases in ROS, implicating a role for FAO in driving this process (126).

Compared to effector cells, CD8 memory T cells (Tmem) are less glycolytic and demonstrate a much higher SRC, implying a greater dependence on OXPHOS as a source of cellular energy. The primary driver of OXPHOS in these cells has been suggested to be FAO (127), supported by murine studies that showed that the memory-inducing cytokine IL-15 enhanced mitochondrial biogenesis and increased expression of the FAO enzyme CPT1A (128). Notably, inhibiting glycolysis at the point of CD8 memory cell formation improves CD8 memory cell function and generates effective antitumor effector cells upon secondary expansion (129). Other work has demonstrated that increasing AMPK signaling, or restricting mTOR activity, also increases Tmem formation in mice (20, 130). This aligns with the fact that overexpression of PPARγ coactivator 1-alpha (PGC-1α), a regulator of mitochondrial biogenesis, increases the generation of CD8 central memory T cells and, in general, improves murine immune responses following infection, immunization, and in the generation of antitumor immunity (131).

Tumor-infiltrating CD8+ Teff often lose effector function upon establishing residence in the tumor microenvironment (TME). New approaches are being explored to enhance antitumor activity within the TME, keeping in mind the harsh metabolic constraints of the local environment. CD8+ T cells in lipid-rich tumors often upregulate the scavenger receptor CD36, causing cells to become dysfunctional over time through over-accumulation of lipid species and subsequent lipid peroxidation (132, 133). One novel approach to mitigate these effects involves metabolically reprogramming CD8+ T cells. For example, inhibiting p38 signaling in mice, which normally regulates T cell DNA damage responses and memory formation, can rescue cytolytic activity in these dysfunctional cells (134). Increased p38 signaling normally occurs downstream of CD36. Removing CD36 prevents excessive FA uptake, which decreases lipotoxicity and improves cellular dysfunction, allowing CD8 T cells to maintain antitumor activity (133). Interestingly, this mechanism has recently been linked to ferroptosis, the nonapoptotic form of cell death long implicated as a driver of T cell death in the TME in both humans and mice. Further, ferroptosis has been shown to correlate with poor immunotherapeutic outcomes in patients (135). Whether or not FA uptake and associated lipotoxicity applies to all tumor environments, or only those with an excessive abundance of regional lipid content, remains to be determined. Furthermore, whether enhancing FAO in cells normally destined to accumulate toxic levels of lipid is of therapeutic benefit remains an area of active investigation (136).

Recent discoveries shed additional light on the potential role of FAO within specific CD8+ TME subsets. Adoptive transfer of tumor-specific murine Tc9 cells, so named due to copious secretion of IL-9 (137, 138), elicited much stronger responses against advanced tumors than conventional Tbet^Hi^ Tc1 cells (139, 140). Further, analysis in both mouse models and patient samples determined that tumor-infiltrating Tc9 cells underwent significantly less lipid peroxidation than Tc1 cells, a trait that strongly correlated with their *in vivo* persistence. At the same time, RNA sequencing demonstrated an increase in FAO gene ontology networks, including increased expression of CPT1A. Additionally, free FA levels, particularly of polyunsaturated fatty acids (PUFAs), were lower in Tc9 cells compared to Tc1 cells and increases in FAO positively correlated with increased persistence and survival of tumor-targeting Tc9 cells. Mechanistically, increased CPT1a and subsequent elevation of FAO was mediated by STAT3 signaling, resulting in decreased ROS production and lower levels of lipid peroxidation. Inhibition of FAO with etomoxir diminished the elevated OCR seen in tumor-targeting Tc9 cells and impeded their anti-tumor effects, again linking FAO to heightened mitochondrial respiration and anti-tumor potential (141).

While not dependent upon lipid oxidation, pre-exposure to linoleic acid (LA) also improved the cancer-targeting potential of adoptive T-cell therapies. Linoleic acid (C18:2n-6) is the most abundant ω-6 PUFA (142) and drives multiple essential functions in health and disease (143). Exposure of T cells to LA improved metabolic fitness, prevented exhaustion, and facilitated the development of a memory-like phenotype with superior effector functions. Mechanistically, LA treatment enhanced the formation of contacts between the endoplasmic reticulum and mitochondria in T cells, in turn promoting calcium (Ca2+) signaling and enhancing mitochondrial energetics in both human and mouse cytolytic T cells (144). Together, these changes improved the antitumor potency of CD8 T cells both *in vitro* and *in vivo*, suggesting that pre-exposure to LA could serve as a potent adjunct to adoptive cellular therapies outside of directly impacting FAO.

### Treg

Another critical group of T cells shown to rely on FAO is Tregs. Tregs comprise a unique and heterogeneous subset of T cells whose primary responsibility is suppressing immune responses through direct cell-to-cell contact and/or the secretion of inhibitory cytokines. These actions are beneficial in preventing and treating autoimmune diseases, dampening allergic responses, and reducing rejection following blood and marrow and solid organ transplantation (145, 146). However, in the case of cancer, an abundance of Treg in the TME can facilitate tumor growth by suppressing the function of tumor-killing cytolytic T cells. Thus, modulating Treg to control immune responses is a popular area of therapeutic interest. Treg are defined by expression of the transcription factor forkhead box P3 (FoxP3) and exhibit a canonical shift in metabolism, prioritizing FAO and OXPHOS over glycolysis in both mice and human cells (147–149). Indeed, in murine studies, selecting cells with high levels of FoxP3 following culture in Treg favorable conditions yielded T cells with increased OCR and an improved ability to function in environments with low glucose (150). Mechanistically, the fatty acid binding protein (FABP), FABP5, was found to be upregulated in these Treg. Importantly FABP5 is a key facilitator of transporting fatty acids across the cell membrane. Inhibition of FABP5, in human or murine Treg, either pharmacologically or through genetic elimination, leads to loss of mitochondrial structure and a reduction in fatty metabolism, both of which together compromise Treg suppressive capacity (151).

More recent work found that signaling through aryl hydrocarbon receptors (AhR) promotes Treg generation (152). Specifically, the treatment of naive murine CD4 T cells with two independent AHR agonists upregulated the expression of FAO-related enzymes, including CPT1a and ACADL, and simultaneously increased OCR in these cells. Genetic or pharmacological inhibition of FAO markedly diminished these changes and subsequently impaired the degree of Treg differentiation. AHR agonists functioned by upregulating mRNA expression of Skp2, an enzyme important in ubiquitination. Mechanistically, the promotion of K63 ubiquitination on liver kinase B1 (Lkb1) facilitated Lkb1-Strad-Mo25 complex formation and activated Lkb1 upstream of AMPK, which then promoted FAO (153). This increase in Skp2-mediated Lkb1 ubiquitination, with a subsequent increase in FAO activity, was verified *in vivo* in Treg recovered from a dextran sodium sulfate-induced colitis murine model. Together, these findings demonstrate that AhR signaling promotes Treg generation concurrent with enhanced Lkb1-mediated FAO, suggesting that AhR agonists might be leveraged therapeutically as potent inducers of Treg cells to prevent and treat autoimmune disease (153).

Although not directly related to FAO, lipid peroxidation has also been studied in intratumoral Tregs. Treg-specific deletion of glutathione peroxidase 4 (Gpx4) impaired immune homeostasis without affecting steady-state Treg survival. However, upon TCR/CD28 co-stimulation of murine Treg, loss of Gpx4 resulted in excessive lipid peroxidation and subsequent ferroptosis. Neutralization of lipid peroxidases and blockade of iron availability rescued ferroptosis in Gpx4-deficient Treg cells. Functionally, loss of Treg cells secondary to Gpx4 ablation potentiated antitumor activity, establishing a critical role for Gpx4 in protecting intratumoral Treg and offering a novel therapeutic strategy to improve anti-cancer treatments (154).

The methods by which Treg can be metabolically manipulated are also evolving. Whole organism delivery of cell therapies and targeted therapeutics lacks tissue-specific localization, allowing for the possibility of unwanted systemic side effects. Further, in the case of spatially restricted autoimmune diseases like psoriasis, systemic delivery creates a situation in which the therapeutic product (e.g. a FAO modulator) may not build up to sufficient quantities in the local environment to be efficacious. Specifically to Treg, systemic administration can limit Treg accumulation in pathologic sites of inflammation within the skin (e.g., the epidermis) due to minimal expression of skin-homing receptors on the migrating Treg (155, 156). Instability of the Treg phenotype, as well as functional T cell loss, are additional critical issues that continue to impede clinical translation. Fortunately, these challenges can be addressed through local delivery of cells combined with regional application of metabolic modulators. Treatment of psoriasis using perforated microneedles (PMN) facilitated Treg delivery directly to the local site of skin disease in animal models and *ex vivo* human samples, effectively administering a local suppressive therapy with regional Treg survival (157). This PMN platform could also be used to release short-chain FAs (SCFAs) into the hyperinflammatory area, further enhancing Treg suppressive functions. Given that Treg administered using PMN technology ameliorated signs and symptoms of psoriasis in a mouse models suggests that localized delivery of both the cell product and metabolic agents could improve the outcomes of adoptive cell therapies (157). This innovation offers a potentially transformative approach to facilitate local treatment while maintaining the phenotypic stability of the transferred product and limiting off-target or on-target/off-tissue effects.

## Conclusion

FAO has diverse roles in the immune system, including between cell compartments and even within individual cell types (summarized in **Tables 1** and **2**). Metabolic choices also evolve as cells differentiate and move through different *in vivo* environments. As highlighted above, FAO can both promote inflammation within ILC2s in the lungs but also simultaneously drive anti-inflammatory activity in tolerogenic DCs in the TME. Similarly, different cell types within the same microenvironment utilize FAO to opposite ends; with FAO promoting the inflammatory function of TME-associated Tc9 cells while encouraging the suppressive function of Tregs. FAO can even create contradictory profiles within the same cell type, with evidence that the anti-inflammatory activity in M2 macrophages is promoted by FAO, as is the pro-inflammatory macrophage function driven by CD40 agonism. These data highlight the complexity of correlating metabolic pathway utilization to specific immune cell functions, particularly as there is not a one-to-one correlation between pathway use and a defined functional output. This nuanced understanding also speaks to the metabolic flexibility of immune cells and further highlights the need to continue to define compensatory metabolic responses engaged upon primary pathway inhibition or blockade. Thus, studies focusing on context-specific utilization of FAO and the subsequent impact of its manipulation represent the best way to uncover new roles for this fundamental, energy-producing pathway. Only in this manner will we fully utilize our knowledge of metabolic reprogramming to attain the most therapeutic immune response.

**Table 1:** Innate immune cell metabolism.

Innate Immune Cells			
Primary Cell Type	Cell subsets	Metabolic Processes	References
Innate Lymphoid Cells	Non-cytolytic: Innate lymphoid cell 1 (ILC1) Innate lymphoid cell 2 (ILC2) Innate lymphoid cell 3 (ILC3)/ Lymphoid Tissue Inducers (LTi) Cytolytic: Natural Killer cells (NK)	Gly FAO/OXPHOS, LM/LDF, Arginine catabolism, Gly Gly/OXPHOS, Vitamin A metabolism Gly, AAM, FAO	37 37, 37-42 37 43-45
Dendritic Cells	Conventional: Conventional dendritic cell 1 (cDC1) Conventional dendritic cell 2 (cDC2) Conventional dendritic cell 3 (cDC3) Tolerogenic DCs Plasmacytoid DCs (pDCs) Inflammatory DCs Langerhans cells Mononuclear Phagocytes (tumor)	Na: OXPHOS, Ac: GU, Gly, PPP, Dys: LDF Na: OXPHOS, Ac: GU, Gly, PPP, Dys: LDF Not reported FAO/OXPHOS Gly/OXPHOS/FAO Gly Na/Ac/Ma: Not reported Dys: LDF Na: OXPHOS, Ac: Gly, PPP In tumors/Dys: LM/LDF	49, 52-53, 55, 66 49, 52-53, 55, 66 52, 57, 61-69 56 52, 55 48 67-72
Macrophages	Inflammatory "M1" macrophage Reparative "M2" macrophage Tumor Associated macrophage (TAM)	Na: OXPHOS, Ac: GU, Gly, PPP. Arg catabolism Na: FAO/OXPHOS, Ac: FAO/OXPHOS, Gly, PPP. Upregulates Arg1 Na: OXPHOS, Ac: GU, Gly, Tumor/Dys: FAO, LM/LDF	72, 79, 82, 84 72, 79, 82-87 84, 88-93

**Table 2:** Adaptive immune cell metabolism.

Adaptive Immune Cells			
Primary Cell Type	Cell subsets	Metabolic Processes	References
B cells	B cell 1 (Innate-like) (B1) B cell 2 (Follicular) (B2) Germinal center B cells (GCBCs) B regulatory cell (Breg)	GU, Gly/OXPHOS, PPP, FAS, LM/LDF Na: FAO/OXPHOS, Ac: GU, Gly, PPP. Ma: GU, AAM, Dys (autoreactive): LM, FAO (ACh-dep)/OXPHOS FAO/OXPHOS Unclear. Implications with HIF-1a and SCFAs	95-97 98-103, 109-110 104-109 101
T cells	Thymocytes Naïve Activated T cells (general) T Helper 1 (TH1) T Helper 2 (TH2) T Helper 17 (TH17) CD 8 Cytolytic Tc9 (tumor) Memory T cells T regulatory (Tregs)	Gly, Glm, FAO/OXPHOS FAO/OXPHOS, AAM GU, Gly GU, Gly, GtU, AAM GU, Gly GU, Gly GU, Gly. Dys (tumor): LM/LDF. Alloreactive: FAO FAO/OXPHOS FAO/OXPHOS FAO/OXPHOS. Dys: LDF	112-117 13-15, 20-24, 120, 123, 125, 147-148 13-15, 24, 120-121 13-14, 16, 18- 24, 120-121, 124-130, 132- 136, 142-144 139-141 20-24, 127-131 149-157

Na, Naïve; Ac, Activated; Ma, Maturation; Mem, Memory; Dys, Dysfunctional; Gly, Glycolysis; Glm, Glutaminolysis; OXPHOS, Oxidative Phosphorylation; FAO, Fatty Acid Oxidation; FAS, Fatty Acid Synthesis; GU, Glucose Uptake; GtU, Glutamine Uptake; PPP, Pentose Phosphate Pathway; LM, Lipid Metabolism; LDF, Lipid Droplet formation; AAM, Amino Acid metabolism.

## Author contributions

FK: Conceptualization, Writing – original draft, Writing – review & editing. EB: Writing – review & editing. CB: Conceptualization, Funding acquisition, Supervision, Writing – original draft, Writing – review & editing.
